# The burden of vitamin D deficiency in household members of children presenting with symptomatic vitamin D deficiency

**DOI:** 10.3389/fendo.2022.958422

**Published:** 2022-08-30

**Authors:** Suma Uday, Wolfgang Högler

**Affiliations:** ^1^ Department of Endocrinology and Diabetes, Birmingham Women’s and Children’s Hospital, Birmingham, United Kingdom; ^2^ Institute of Metabolism and Systems Research, University of Birmingham, Birmingham, United Kingdom; ^3^ Department of Paediatrics and Adolescent Medicine, Johannes Kepler University Linz, Linz, Austria

**Keywords:** household, hidden burden, micronutrient deficiency, folate, hypovitaminosis

## Abstract

**Background:**

Micronutrient deficiencies are common among household/family members due to shared lifestyle and dietary habits. The extent of biochemical abnormalities in household members of children presenting with symptomatic vitamin D deficiency remains unknown.

**Aim:**

Investigate the prevalence of vitamin D deficiency and biochemical osteomalacia in the mothers and siblings of children presenting with symptomatic vitamin D deficiency.

**Methods:**

All mothers and sibling of children referred to a single tertiary endocrine centre between January 2018 and December 2021, with symptomatic vitamin D deficiency were investigated prospectively for vitamin D deficiency [defined as 25 hydroxyvitamin D (25OHD) < 30nmol/L] and biochemical osteomalacia [vitamin D deficiency and elevated alkaline phosphatase (ALP) and/or parathormone (PTH)] as per clinical guidelines.

**Reults:**

Ninety-seven family members (68 siblings and 29 mothers) of 29 index cases (median age 1.7 years, 55.5% male) were investigated. The majority (65.5%, n=19) were of Asian ethnic background. The mean (SD) 25OHD levels of the index, maternal and sibling cohorts were 15 (10), 15 (7) and 20 (10) nmol/L respectively. Vitamin D deficiency was noted in 93% of the maternal and 79% of the sibling cohorts. Biochemical osteomalacia was present in 72% of the maternal and 79% of the sibling cohorts. Mothers of infants had significantly lower mean 25OHD levels compared to mothers of older children [11 (n=12) vs 18 nmol/L (n=17) respectively, p=0.006)], most of whom were symptomatic (66.6%, n=8/12). None of the mothers had hypocalcaemia. Among the 10% (n=7) of the siblings with hypocalcaemia, 86% (n=6/7) had concurrent dietary calcium deficiency and 71.4% (n= 5/7) reported symptoms in retrospect. Hypocalcaemic siblings had significantly lower 25OHD (7 vs 15 nmol/L, p<0.001), higher PTH (175 vs 58 ng/L, p<0.001) and ALP (846 vs 318 IU/L, p<0.001), respectively compared to normocalcaemic siblings.

**Conclusions:**

In view of the substantial morbidity uncovered in household/family members of children diagnosed with symptomatic vitamin D deficiency, we recommend universal supplementation of all risk groups. Biochemical testing and treatment is indicated to replenish stores only in those at highest risk such as mothers of infants, individuals with concurrent dietary calcium deficiency and those with clinical symptoms.

## Introduction

Micronutrient intake tends to be similar between household members and there is evidence that poor diet in parents, especially the mother, is strongly correlated with poor diet in the offspring (1). Micronutrient deficiencies in the offspring are reported to be strongly influenced by maternal education levels (2). Similarly, supplement uptake, such as vitamin D uptake in children has been shown to be influenced by maternal factors such as age and education (3). In high latitude countries with reduced Ultraviolet B (UVB) radiation vitamin D deficiency is very common given that its main source is skin synthesis following UVB exposure (4). Hence, reliance on vitamin D supplements for adequacy is high. National vitamin D supplementation guidance, especially in high risk groups such as dark skinned individuals, exist in the majority of high latitude countries (5) including the UK (6, 7). The uptake of vitamins however is very variable (5) which has led to the resurgence of nutritional rickets in several high latitude countries (8), especially where food fortification with vitamin D is not mandated.

Deficiency in vitamin D remains one of the most common causes of nutritional rickets worldwide (9, 10). Vitamin D deficiency leads to reduced calcium absorption from the gut, which reduces mineral supply for bone mineralization. Reduced bone mineralization (osteomalacia) affects adults and children and children additionally develop the growth plate disorder rickets (11). Rickets not only presents with bony deformities, fractures, stunted growth and delayed development (12) but can also manifest with hypocalcaemic complications which include seizures, tetany and rarely heart failure due to dilated cardiomyopathy (13–15). A series of UK studies have described the clinical presentation (16, 17), incidence (15, 18) and complications (13, 14) related to vitamin D deficiency in children but none have systematically explored the hidden burden in their family members. We have previously uncovered a high proportion of vitamin D deficiency in family members of three infants presenting with life-threatening features of vitamin D deficiency (14). In the current report we examine the unexposed burden of vitamin D deficiency in apparently healthy family members of index children presenting to hospital with symptomatic vitamin D deficiency.

## Aim

Report the prevalence of vitamin D deficiency and biochemical osteomalacia in mothers and siblings of children presenting with symptomatic vitamin D deficiency.

## Methods

Biochemical evaluation of household members (mother and siblings) of children presenting with symptomatic vitamin D deficiency, at a single tertiary endocrine centre between January 2018 and December 2021, was undertaken prospectively.

### Subjects

Household members of all children referred to the paediatric endocrine team at Birmingham Women’s and Children’s Hospital (BWCH) for management of symptomatic vitamin D deficiency were included.

### Study period

The study was conducted between January 2018 and December 2021 (4 years).

### Setting

In accordance with the trust guidelines only cases with significant symptoms (seizures, tetany, bone deformities, fractures) and infants are routinely discussed with the endocrine team. Older children without complications (identified to have vitamin D deficiency on routine bloods) are managed by other teams as per the guidance.

### Ethics

Ethical approval was not required as investigations were undertaken as standard of care provided. The work was registered as a clinical management audit and data gathered in accordance with the trust guidance.

### Data collection

Clinical, laboratory [serum adjusted calcium, phosphate, 25 hydroxyvitamin D (25OHD), parathormone (PTH), alkaline phosphatase (ALP), haemoglobin and Mean Cell Volume (MCV)] and radiological data, from routine clinical care, were collected prospectively on all subjects presenting with symptomatic vitamin D deficiency during the study period.

Biochemical evaluation of vitamin D deficiency (25OHD, calcium, phosphate, ALP, PTH) is routinely recommended for all mothers and siblings of the index case and these data were gathered. Further investigations in the siblings such as radiographs were undertaken based on clinical need and where available this data was gathered.

### Classification

#### Vitamin D deficiency

Deficiency was classed as 25OHD concentrations <30 nmol/L (12 µg/l), insufficiency as 30-50 nmol/L (12-20 µg/l) and sufficiency >50 nmol/L (>20 µg/l) based on the Institute Of Medicine (19) and Global consensus recommendation classifications (20). Levels <25 nmol/L (10 µg/l) are also reported as this is the commonly used cut off in the UK as per the Scientific Advisory Committee on Nutrition (SACN) recommendations (21).

#### Biochemical osteomalacia

Several definitions of biochemical osteomalacia have been proposed (22, 23) and for the purpose of this work it was defined as vitamin D deficiency combined with elevated alkaline phosphatase (ALP) and/or elevated parathormone (PTH) (22, 23). As normocalcaemia is maintained through compensatory mechanisms until the late stages (24, 25) it was not included as a requirement for biochemical osteomalacia.

### Laboratory methods

Serum 25OHD concentrations were measured by quantitative liquid chromatography tandem mass spectrometry (AB Sciex API4000 MS/MS’ analyser) before 12/05/2020 and by Radio Immuno Assay (Roche Cobas C6000) after 12/5/2020. The laboratory is subject to External Quality Assurance (EQA) and meets the requirement of the UK NEQAS (National External Quality Assessment Service) vitamin D scheme. The inter and intra assay CVs were < 10% for both methodologies.

Serum ALP activities were determined using a dye-based assay, which measures the enzyme activity by monitoring the rate of hydrolysis of p-nitrophenylphosphate to p-nitrophenol at 410/480 nm in the presence of magnesium on the 182 Olympus AU640 analyser.

Intact PTH was measured using the Immulite 2000 PTH assay (Siemens Healthcare Diagnostics Products, Erlangen, Germany) which is a solid-phase, two-site chemiluminescence enzyme-linked immunosorbent assay. The reference ranges for ALP and PTH were derived from a Combination of CALIPER reference ranges (26) and assay specific reference range based on manufacturer recommendations.

### Statistics

Descriptive statistics are presented as median or mean with standard deviation (SD) for continuous variables, and frequencies with percentages for categorical variables. A two-tailed independent sample t-test was used to evaluate the difference in 25OHD levels between groups. All statistical analyses were performed using SPSS statistical software v28.0 (IBM, Armonk, NY, USA).

## Results

A total of 97 family members (29 mothers and 68 siblings) of 29 index cases were investigated.

### Index cohort

**Figure 1:**
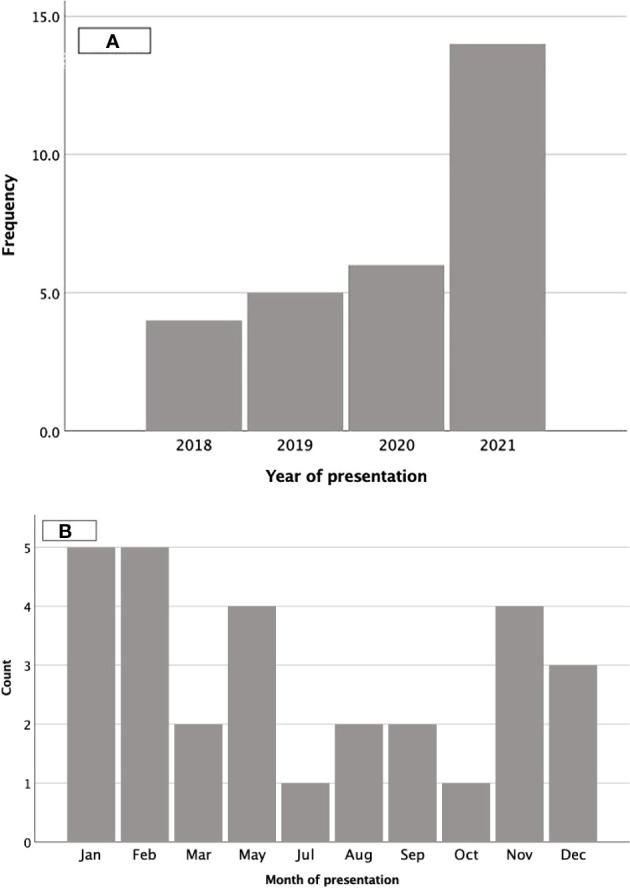
Presentation by calendar year and month. **(A)** represents the number of children presenting by year and **(B)** by month.

The median age of the index patient cohort was 1.7 years (range 6 days-15 years, of the 29 subjects 12 were infants and 7 adolescents). The majority were male (n=16, 55.5%). The highest number of cases were recorded in 2021 (48.2%, n=14, **Figure 1A**) and they frequently presented between November and February (58.6%, n=17, **Figure 1B**). The majority were of Asian (65.5%, n=19) and black African-Caribbean (24.1%, n=7) background, the remainder (one each) were of Somali, White British, and mixed Asian and white British background. Two of the three adolescent girls practiced religious covered clothing. The clinical presentation is detailed below (**Box 1**) and the biochemical findings are presented in **Table 1**.

Box 1

**Table:** 

Presenting feature	n (%)
Hypocalcaemic Seizures	11 (37.9)
Bowing of legs and short stature	4 (13.8)
Delayed development or regression of milestones	3 (10.3)
Poor appetite and non-specific feature of tiredness	3 (10.3)
Breathlessness and poor feeding	3 (10.3)
Fractures	2 (6.9)
Identified on dilated cardiomyopathy screen	1 (3.4)
Incidental hypocalcaemia on bloods done for other indications (1= bowing of legs present, 1= suspected previous seizure like episodes)	2 (6.9)

**Table 1:** Biochemical parameters in the index, maternal and sibling cohorts.

	Serum Adjusted Calcium mmol/L	Serum phosphate mmol/L	Serum Alkaline Phosphatase IU/L	Serum 25 hydroxy vitamin D nmol/L	Serum Parathormone ng/L
Normal range adults	2.2 - 2.6	0.8 - 1.5	25 – 105	>50	15 – 65
Normal range Paediatric	2.2 - 2.7	0.9 - 1.8	Age and gender (M=male, F=female) specific *	>50	13 – 29
Index cohort (n=29)					
Mean ± SD Median (range)	1.79 (0.38) 1.78 (1.16-2.50)	1.41 (0.73) 1.21 (0.47-3.39)	952 (858) 774 (114-4092)	15 (10) 12 (5-49) <7.5 in n=8	299 (269) 238 (31-1345)
Maternal cohort (n=29)					
Mean ± SD Median (range)	2.28 (0.06) 2.3 (2.25-2.47)	1.3 (0.10) 1.2 (0.9-1.3)	108 (37) 90 (77-180)	15 (7) 13 (<7.5-33) <7.5 in n=2	81 (19) 85 (54-110)
Sibling cohort (n=68)					
Mean ± SD Median (range)	2.23 (0.19) 2.3 (1.75-2.47)	1.36 (0.23) 2.29 (0.74-1.69)	411 (423) 353 (97-2455)	20 (10) 19 (7-45)	83 (73) 60 (19-301)

*0- 4wk: 50-230, 5wk-9y: 80 – 330, 10-11 y (F:80-310, M:80-240)12-13y (F:65-240, M:90-290), 14-15y (F:55-150, M:55-320), 16-18y (F:25-90, M:40-240).

None of the index cases were on vitamin D supplements at the time of presentation. All except three subjects had no significant past medical history or co-morbidity (1= urethral valve, 1= known epilepsy presented with increased seizure frequency, 1= congenital cyst in the liver).

Dietary history indicated concurrent dietary calcium deficiency secondary to dairy avoidance (no medical indication) in three subjects. Two of the three subjects were adolescents and followed a vegan diet (who had a calcium intake of 260 mg and 220 mg daily each). One of the three subjects aged 3 years, referred with bowed legs, avoided dairy products due to health beliefs (who had a calcium intake of 320 mg daily), and had been treated for vitamin D deficiency in the past by the general practitioner but was not recommended further ongoing supplementation.

The biochemical data for the cohort is presented in **Table 1**. All except two individuals had 25OHD levels < 30 nmol/L. The clinical presentation, age and laboratory parameters of the two children with 25OHD levels > 30 nmol/L and suspected to have concurrent dietary calcium deficiency are presented below (**Box 2**).

Box 2

**Table:** 

Presentation	Calciummmol/L(2.2-2.7)	Phosphate mmol/L(0.9–1.8)	ALPIU/L*	PTH ng/L (13-29)	25OHD nmol/L(>50)
Dilated cardiomyopathy screening (6 months)	2.2	1.2	1060	362	48.9
Hypocalcaemic seizure (day 6 of life)	1.74	3.39	530	61	34.3

* 0- 4wk: 50-230 IU/L, 5wk-9y: 80 – 330 IU/L.

Two of the three infants who presented with breathlessness and poor feeding had cardiac dysfunction [1= Dilated cardiomyopathy (DCM) and reduced left ventricular (LV) function, 1= reduced LV function]. All three infants with DCM were screened for genetic causes but no mutations were identified. Reversal of LV dysfunction was documented in all subjects following treatment with cholecalciferol and calcium supplements. Radiological evidence of rickets (fraying, splaying and cupping) at the knee and/or wrist was present in sixteen of the twenty-one subjects (76.2%) who had radiographs.

Low haemoglobin with or without low Mean Cell Volume was noted in 44.8% (n=13/29) and 13.8% (4/29) respectively. Two (of the 29) individuals also had folate and vitamin B12 deficiency.

### Maternal cohort

**Figure 2:**
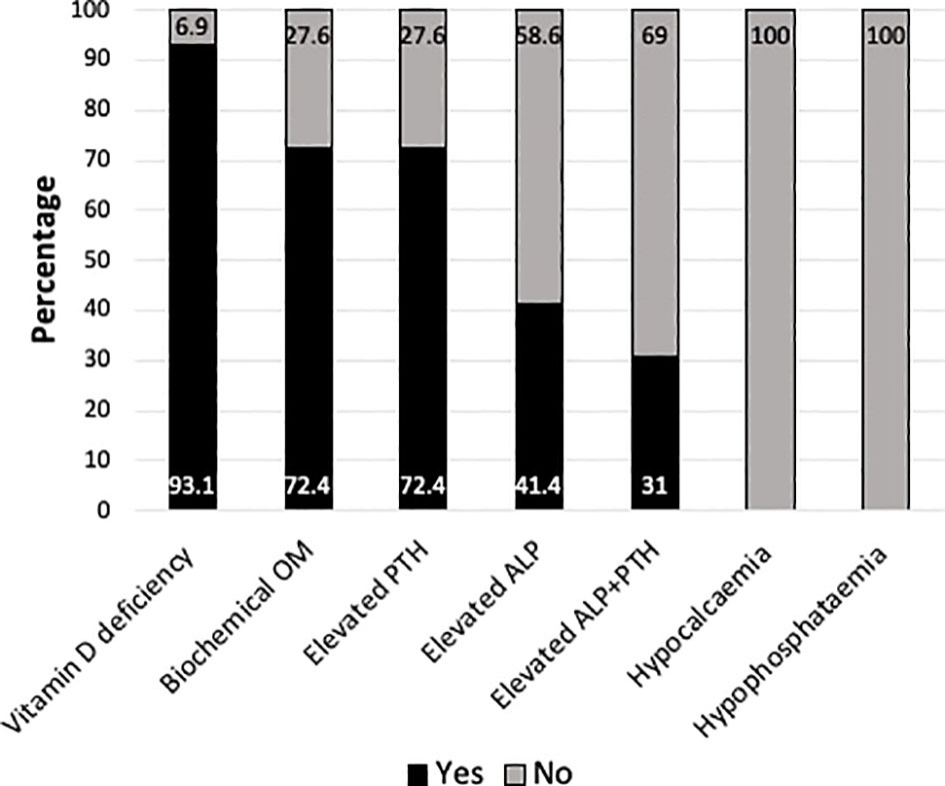
Bar chart representing the proportion of mothers with vitamin D deficiency (25hydroxyvitamin D <30 nmol/L) (n=27/29), biochemical osteomalacia (OM)* (n=21/27), elevated parathormone (PTH)(n=21/29) or alkaline phosphatase (ALP)(n=12/29) or both (n=9/29), hypocalcaemia (n=0/29) and hypophosphataemia (n=0/29). *Biochemical OM was defined as 25OHD <30 nmol/L + elevated ALP and/or PTH.

The biochemical data are presented in **Table 1** and the proportion with biochemical abnormalities is illustrated in **Figure 2**. The median (range) age of the maternal cohort was 28.3 (23.4 – 38.9) years. All except two mothers (31 and 33 nmol/L) had 25OHD levels < 30 nmol/L (93.1%, 27/29) of whom 88.9% (n=24/27) had levels < 25 nmol/L. A total of six mothers practiced religious covered clothing. Mothers with affected infants (n=12) had significantly lower 25OHD compared to those with affected older (n=17) children (mean 11 nmol/L vs 18 nmol/L respectively, p=0.006) most of whom were symptomatic (66.6%, n=8/12). Three of the four women who had the lowest 25OHD levels (< 10 nmol/L) were breastfeeding, two were mothers of infants (aged 6 days and 3 months) and one was prolonged breastfeeding at the age of two years. None of the mothers were on supplements at the time of presentation but six reported taking supplements during pregnancy and three were treated for vitamin D deficiency in the past (not during pregnancy).

### Sibling cohort

**Figure 3:**
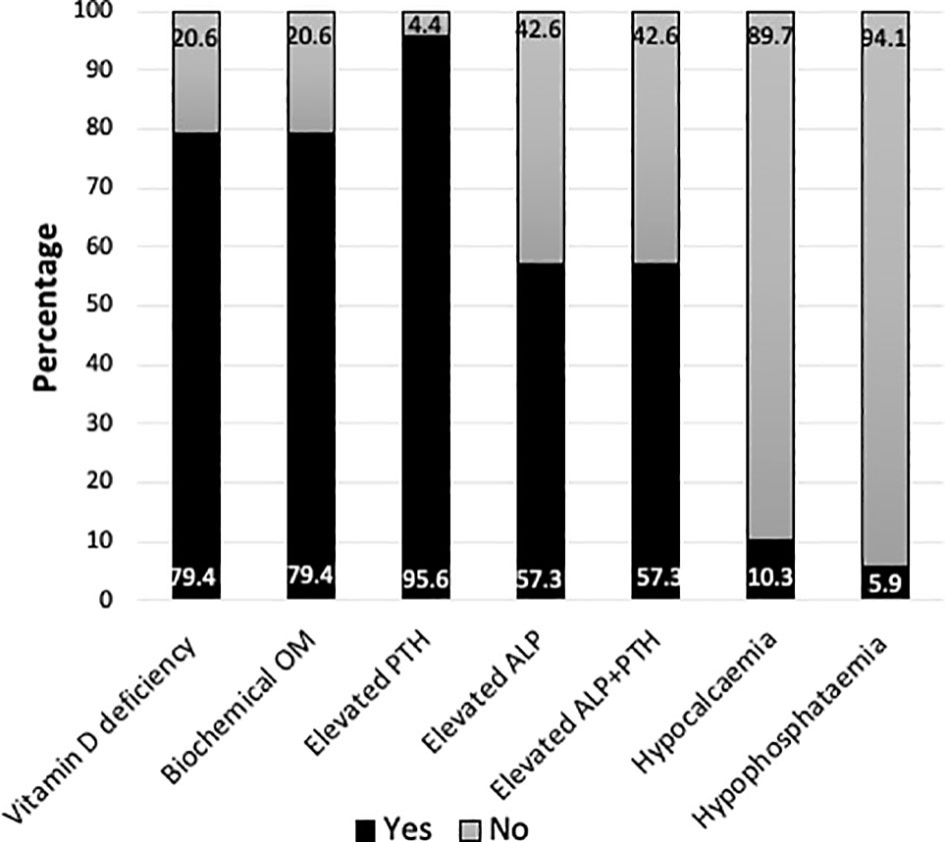
Bar chart representing the proportion of siblings with vitamin D deficiency (25hydroxyvitamin D <30 nmol/L) (n=54/68), biochemical osteomalacia (OM)* (n=54/68), elevated parathormone (PTH)(n=65/68) or alkaline phosphatase (ALP)(n=39/68) or both (n=39/68), hypocalcaemia (n=7/68) and hypophosphataemia (n=4/68). *Biochemical OM was defined as 25OHD <30 nmol/L + elevated ALP and/or PTH.

The median (range) age of the sibling cohort was 6.6 (1.3-15) years and 58.8% (n=40) were male. The biochemical data are presented in **Table 1** and the proportion of children with biochemical abnormalities are illustrated in **Figure 3**. The majority were vitamin D deficient (79.4%, n=54) and the rest insufficient (20.6%, n=14). All except three subjects, who were on ‘Healthy start’ vitamins, had elevated PTH (95.6%, n=65). Seven (10.3%) individuals (from five families) were found to have hypocalcaemia, requiring intervention (n=2 intravenous calcium gluconate and n=5 oral calcium supplements), of whom 71.4% (n= 5/7) reported symptoms in retrospect. The median age of siblings with hypocalcaemia was 9 years (range 1.3-11 years) and they had significantly lower mean 25OHD (7 vs 15 nmol/L respectively, p<0.001) and a higher mean PTH (175 vs 58 ng/L respectively, p<0.001) and ALP (846 vs 318 IU/L respectively, p<0.001) when compared to the siblings without hypocalcaemia. Concurrent calcium deficiency was noted in 85.7% (n=6/7) of the siblings with hypocalcaemia. Calcium deficiency due to complete dairy avoidance (for non-medical reason) was recorded in two of the subjects from the same family who also had severe vitamin D deficiency (25OHD of 6.8 and 7.4 nmol/L). Four subjects had inadequate dietary calcium intake but not deliberate avoidance. Radiological rickets was recorded in two of the four subjects, aged 1.5 years and 3 years, who had knee radiographs.

## Discussion

This study identified a very high proportion of vitamin D deficiency and biochemical osteomalacia in household/family members of children presenting with symptomatic vitamin D deficiency. All but one family were from ethnic minorities/migration background and 93% of mothers and 79% of siblings had vitamin D deficiency (<30nmol/l or 12 µg/l). Biochemical osteomalacia was present in up to 72% of mothers and 96% of siblings, which underscores the severity of calcium deprivation in these families. Hypocalcaemia was recorded in 10% of the siblings who also had significantly lower 25OHD and higher ALP and PTH when compared to normocalcaemic sibling with vitamin D deficiency. Mothers of infants had significantly lower 25OHD levels compared to mothers of older children demonstrating the typical vertical transmission of micronutrient deficiencies (from the mother to the foetus) and the direct consequences of lack of antenatal vitamin D supplementation on the vitamin D status of the infant (27).

The clinical presentation of vitamin D deficiency (16, 17) and seasonal variation (13, 15) in our cohort was similar to previous reports. A higher number of patients with symptomatic deficiency were seen in 2021. There is anecdotal evidence that cases of vitamin D deficiency have increased following COVID lockdown restrictions, however we did not aim to evaluate the incidence pre and post COVID. The higher numbers seen may be due to the higher number of patients seen in our trust during this period due to mutual aid and ambulance divert. Therefore, increased incidence cannot be concluded.

Two subjects were found to be symptomatic in the absence of significant vitamin D deficiency and in these patients a concurrent calcium deficiency was suspected. Both of these patients were infants. It is known that up to 60% of 25OHD2 or 25OHD3 can exist in its C-3 epimer form in infants which can lead to overestimation of 25OHD levels (28). Commonly available assays, including the assay used here, do not differentiate between 25OHD and its C-3 epimers. It is recommended that serum 25OHD in children below the age of one is measured with an assay that either does not cross-react with C3-epi-25OHD or allows unequivocal separation of C3-epi-25OHD from 25OHD (29). Such assays however, are not widely available in clinical care and restricted for research purposes.

One child with known epilepsy presented with increased seizure frequency and was found to have hypocalcaemia with vitamin D deficiency which was resolved with treatment. Anti-epileptic medication use has been linked to Vitamin D deficiency (30). It is therefore important to monitor serum 25OHD levels in children with chronic health conditions and prevent symptomatic deficiency through optimum supplementation (31).

None of the family members in this study had sufficient vitamin D levels (>50 nmol/L or >20 µg/l). The demographic area covered (West Midlands, UK) has a larger proportion of ethnic minority population who are at a very high risk of vitamin D deficiency (32). Whilst skin colour and dietary calcium intake determines the risk of deficiency, the higher proportion of osteomalacia in family members in our cohort may possibly include an element of genetic predisposition to aberrant vitamin D metabolism. Studying the genes involved in vitamin D metabolism may clarify the role of genetic and non-genetic factors contributing to deficiency states in affected family members. Nonetheless, previous genome wide association studies (n=4501) have demonstrated that polymorphisms in genes encoding key enzymes and proteins that affect the circulating concentrations of vitamin D metabolites only account for less than 5% of the variations in serum 25OHD concentrations (92) with major determinants still being lifestyle related such as exposure to sunlight and dietary habits.

Only a small proportion of women reported taking supplements in pregnancy despite the recommendation that all high-risk pregnant women should be supplemented (33). Whilst routine testing of 25OHD levels is not recommend, dark-skinned pregnant women are in the high-risk category and must be offered testing when symptomatic and treatment as necessary (33). Additionally, women who have been treated for deficiency must be informed of the need for ongoing supplement use not just for themselves but for all family members. Informing pregnant women of the need for supplementation of the newborn, in particular providing written information (34) at discharge from the neonatal units (5), enhances adherence to supplements in infants. Individuals who have been treated for deficiency must be informed of the need for ongoing lifelong supplementation to prevent the risk of recurrence, unless the risk factors can be mitigated (20). It is especially important to educate parents of at-risk populations of the need to supplement all family members. Families who avoid dairy, for non-medical reasons, are at highest risk of multiple members being significantly affected with a higher proportion having hypocalcaemia.

Given the high prevalence of deficiency and biochemical osteomalacia in family members it may be economically more reasonable to supplement all high-risk individuals than testing for deficiency. For similar reasons food fortification with additional supplementation of risk groups in the UK has been deemed to be a cost-effective approach to tackling widespread deficiency (35). In our cohort we noted that certain groups have a particularly higher risk such as mothers of infants and individuals with dietary calcium deficiency. Unless robust antenatal vitamin D supplementation programmes are established it would be crucial to test and treat, and not just supplement, mothers of infants with symptomatic deficiency. Hypocalcaemia was noted in nearly 10% of the sibling cohort, 86% of whom had concurrent dietary calcium deficiency. Hypocalcaemia is known to be a late feature of calcium deprivation (25). If severe deficiencies are left untreated, especially in children, they are likely to proceed to acute presentation with seizures, tetany or muscle cramps (14). The majority of individuals from these higher risk groups were also symptomatic, warranting testing and using treatment doses of vitamin D (and calcium when indicated), not just supplementation doses of vitamin D to replenish the stores before moving on to routine supplementation or prevention doses.

In conclusion, all household family members of children with symptomatic deficiency had deficient or insufficient vitamin D status with a high proportion having biochemical osteomalacia. These findings necessitate a change in clinical practice, specifically to include family members from ethnic minorities in morbidity assessment. Since all family members have low vitamin D status, it would make sense economically to supplement all family members. However, many family members are in fact also symptomatic, so testing and treating those at highest risk i.e mothers of infants, those with concurrent dietary calcium deficiency and symptomatic individuals, is essential to replenish stores to alleviate symptoms and prevent acute clinical presentations. Educating parents of the need to continue lifelong supplementation for the whole family, unless risk factors can be mitigated, constitutes an essential public health intervention in an increasingly diverse population.

## Data availability statement

The raw data supporting the conclusions of this article will be made available by the authors, without undue reservation.

## Ethics statement

Ethical review and approval was not required for the study on human participants in accordance with the local legislation and institutional requirements. Written informed consent to participate in this study was provided by the participants’ legal guardian/next of kin.

## Author contributions

SU: Data gathering, analysis, manuscript preparation, final approval. WH: Conceptualisation, intellectual revision of the content and final approval.

## Acknowledgments

We would like to thank all the clinicians involved in the management of these patients. We would like to extend our special thanks to Dr Ruchi Nadar, Metabolic bone fellow at BWCH, for alerting SU of the cases and facilitating family investigations when necessary.

## Conflict of interest

The authors declare that the research was conducted in the absence of any commercial or financial relationships that could be construed as a potential conflict of interest.

## Publisher’s note

All claims expressed in this article are solely those of the authors and do not necessarily represent those of their affiliated organizations, or those of the publisher, the editors and the reviewers. Any product that may be evaluated in this article, or claim that may be made by its manufacturer, is not guaranteed or endorsed by the publisher.
